# Editorial: Cardiac reverse remodeling after novel heart failure therapies—volume II

**DOI:** 10.3389/fcvm.2024.1528743

**Published:** 2025-01-10

**Authors:** Christian Basile, Elisabetta Salvioni, Stefania Paolillo, Piergiuseppe Agostoni, Massimo Mapelli

**Affiliations:** ^1^ Department of Advanced Biomedical Sciences, University of Naples Federico II, Naples, Italy; ^2^ Department of Clinical Sciences and Community Health, Cardiovascular Section, University of Milan, Milan, Italy; ^3^ ERN GUARD-Heart Center for Diagnosis and Treatment of Cardiomyopathies, Cardiovascular Department, ASUGI, University of Trieste, Trieste, Italy

**Keywords:** heart failure, heart failure prognosis, cardiac reverse remodeling, heart failure therapies, ejection fraction

**Editorial on the Research Topic**
Cardiac reverse remodeling after novel heart failure therapies—volume II

Heart Failure (HF) is a global pandemic accounting for 56 million prevalent cases worldwide (1). It is also a chronic progressive syndrome characterized by a progressive decline in quality of life and functional capacity, accompanied by an increase in morbidity and mortality (2). The progressive decline in cardiac function and structure is considered the main factor associated with declining prognosis in HF (3), leading to reverse remodeling, i.e., the reverse of these pathological processes, being a significant target for HF treatments (4). In this context, the six studies included in this Research Topic contribute to further highlighting how the trajectories of our patients may be diverted from progressive worsening and how observational research can explore settings not formally tested in randomized controlled trials.

The first study by Chan et al. delves into the long-term effects of cardiac reverse remodeling in HF patients receiving novel therapies. Using observational data, this study highlights the predictive value of left ventricular ejection fraction (EF) improvements on patient outcomes. It emphasizes how even modest improvements in EF can yield significant prognostic benefits. Through detailed analysis, Chan et al. underscore the complexity of reverse remodeling and suggest potential biomarkers that could predict which patients are most likely to experience structural cardiac improvements with advanced HF therapies.

Segev et al. present a focused investigation on HF with improved EF (HFimpEF), analyzing patient characteristics, clinical trajectories, and predictors of EF improvement in a cohort of ambulatory HF patients. Their findings suggest that while EF improvement is generally associated with better clinical outcomes, factors such as baseline EF, age, and HF etiology, particularly ischemic heart disease, play a critical role in determining which patients are most likely to experience meaningful reverse remodeling. This study's predictive model, based on clinical and echocardiographic parameters, offers a promising tool for identifying patients who may benefit from targeted HF management strategies.

Yang et al. examine the real-world effectiveness of sacubitril/valsartan in HF patients on dialysis, a group often excluded from clinical trials. Their study reveals that, while sacubitril/valsartan therapy shows similar cardiovascular benefits to angiotensin-converting enzyme inhibitors or angiotensin receptor blockers in this very high cardiovascular risk population, the risks of hypotension and hyperkalemia appear to be lower with sacubitril/valsartan. This real-world evidence supports the safe and potentially beneficial use of sacubitril/valsartan in a challenging subset of HF patients, expanding our understanding of HF treatment options for individuals with end-stage renal disease.

In the study by Cao et al., the impact of surgical strategies on reverse remodeling and outcomes in HF patients with reduced ejection fraction undergoing coronary artery bypass grafting (CABG) is examined. Comparing on-pump and off-pump CABG techniques, Cao et al. find that on-pump CABG results in lower perioperative mortality and superior long-term survival, particularly for patients with significant left ventricular remodeling. This work suggests that the choice of surgical approach should carefully consider the extent of cardiac remodeling, as patients with severe left ventricular dilation may derive greater benefit from on-pump CABG.

Sousa Nunes et al. present a systematic review and meta-analysis assessing left ventricular reverse remodeling after aortic valve replacement (AVR) in patients with severe aortic stenosis. Through a comprehensive analysis of over 11,000 patients, the study reveals significant lower left ventricular mass and diameter, and higher EF, following AVR. These findings support the concept that AVR may effectively induce reverse remodeling, although the extent of remodeling appears to vary based on age, comorbidities, and AVR technique.

Finally, Li et al. investigate the association between different left bundle branch area pacing (LBBAP) modes on electrocardiographic and echocardiographic responses in HF patients. Their study compares left bundle branch trunk pacing, left anterior fascicle pacing, and left posterior fascicle pacing (LPFP), finding no significant differences in depolarization synchrony, repolarization stability, or echocardiographic response among these modes. However, LPFP emerges as the most common mode in HF patients, offering a stable and effective pacing option for this population, potentially facilitating broader adoption of LBBAP in HF management.

As we conclude the second volume of this Research Topic through the reported studies (5), we acknowledge the crucial role of observational research to assess questions that may not be easily answered in randomized controlled trials.

Does reverse remodeling influence the prognostic value of ejection fraction? Is the effectiveness of sacubitril/valsartan consistent in patients in dialysis? Can reverse remodeling influence the result of coronary-artery bypass graft? Is there a difference in reverse remodeling with different left bundle branch pacing models? What are the predictors of improvement in ejection fraction? Is there reverse remodeling after aortic valve replacement for severe aortic stenosis?

**Figure 1:**
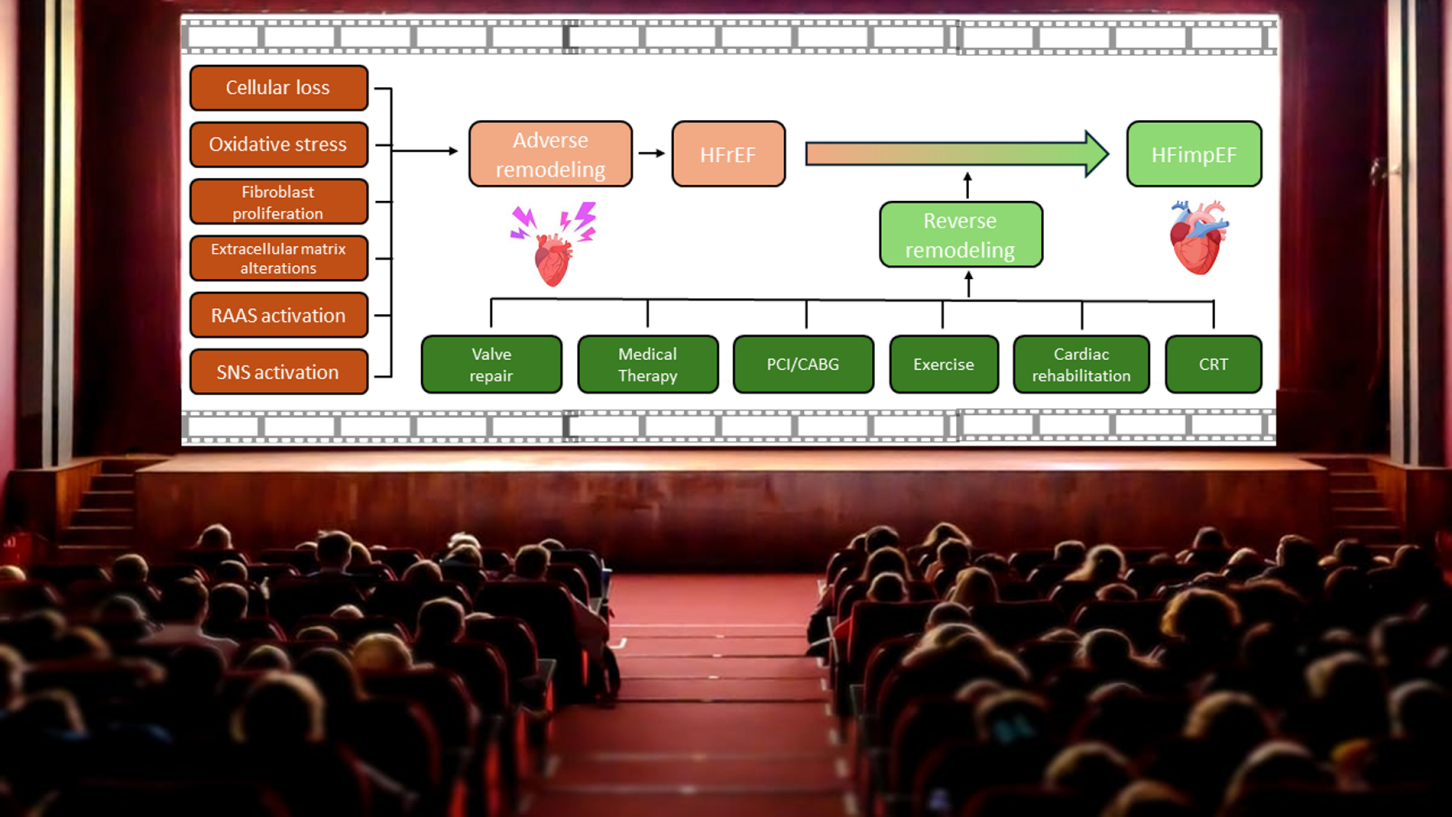
Reverse remodeling, the new actor in the never-ending movie of heart failure management. CRT, cardiac resynchronization therapy; HFimpEF, heart failure with improved ejection fraction; HFrEF, heart failure with reduced ejection fraction; RAAS, renin-angiotensin-aldosterone system; SNS, sympathetic nervous system.

Understanding and discerning the possible interventions to alter the trajectory of HF in our patients is a clear unmet need in our current understanding of HF. Through these studies, we gain deeper insights into patient-specific factors, potential biomarkers, and effective treatment strategies that could transform HF management and improve patient outcomes. Like spectators of a film filled with unexpected, favorable plot twists, our patients watch in awe as therapies for HF can achieve nowadays remarkable reverse remodeling. Being a HF specialist today means rolling up our sleeves, overcoming therapeutic inertia, and stepping up to help direct this film hoping the story leads to a happy ending that restores quality of life and hope to those who need it most (Figure 1).
